# Target-based deep learning network surveillance of non-contrast computed tomography for small infarct core of acute ischemic stroke

**DOI:** 10.3389/fneur.2024.1477811

**Published:** 2024-09-19

**Authors:** Hang Qu, Hui Tang, Dong-yang Gao, Yong-xin Li, Yi Zhao, Qi-qi Ban, Yu-Chen Chen, Lu Lu, Wei Wang

**Affiliations:** ^1^Department of Radiology, Affiliated Hospital of Yangzhou University, Yangzhou, Jiangsu Province, China; ^2^Department of Health Science and Kinesiology, Georgia Southern University, Statesboro, GA, United States; ^3^School of Computer Science and Engineering, Nanjing University of Science and Technology, Nanjing, Jiangsu Province, China; ^4^Chinese Institute of Brain Research, Beijing, China; ^5^Department of Radiology, Nanjing Medical University Affiliated First Hospital, Nanjing, China

**Keywords:** target-based deep learning network, small infarct core, acute ischemic stroke, you only look once (YOLO), non-contrast CT

## Abstract

**Purpose:**

Rapid diagnosis of acute ischemic stroke (AIS) is critical to achieve positive outcomes and prognosis. This study aimed to construct a model to automatically identify the infarct core based on non-contrast-enhanced CT images, especially for small infarcts.

**Methods:**

The baseline CT scans of AIS patients, who had DWI scans obtained within less than 2 h apart, were included in this retrospective study. A modified Target-based deep learning model of YOLOv5 was developed to detect infarctions on CT. Randomly selected CT images were used for testing and evaluated by neuroradiologists and the model, using the DWI as a reference standard. Intraclass correlation coefficient (ICC) and weighted kappa were calculated to assess the agreement. The paired chi-square test was used to compare the diagnostic efficacy of physician groups and automated models in subregions. *p* < 0.05 was considered statistically significant.

**Results:**

Five hundred and eighty four AIS patients were enrolled in total, finally 275 cases were eligible. Modified YOLOv5 perform better with increased precision (0.82), recall (0.81) and mean average precision (0.79) than original YOLOv5. Model showed higher consistency to the DWI-ASPECTS scores (ICC = 0.669, *κ* = 0.447) than neuroradiologists (ICC = 0.452, κ = 0.247). The sensitivity (75.86% vs. 63.79%), specificity (98.87% vs. 95.02%), and accuracy (96.20% vs. 91.40%) were better than neuroradiologists. Automatic model had better diagnostic efficacy than physician diagnosis in the M6 region (*p* = 0.039).

**Conclusion:**

The deep learning model was able to detect small infarct core on CT images more accurately. It provided the infarct portion and extent, which is valuable in assessing the severity of disease and guiding treatment procedures.

## 1 Introduction

Acute ischemic stroke (AIS) is a clinical syndrome of rapid onset of focal cerebral deficit (1), which represents a major public health problem worldwide (2). Since its high mortality, disability and morbidity, emergent diagnosis and treatment is critical for patient prognosis. The current first-choice examination method is non-contrast computed tomography (NCCT) of the head, valued for its speed of acquisition and wide availability (3). However, early AIS does not change significantly on CT, resulting in the previous interpretation of CT signs achieved low sensitivity (40–60%) within the first 3 h after symptom onset (4).

To better assess tissue damage and guide AIS treatments, Alberta Stroke Program Early CT score (ASPECTS) was designed to summarize early signs of ischemia in AIS patients. ASPECTS is a standardized semi-quantitative CT grading system used to quantify early ischemic changes in patients with ischemic changes within 10 regions of the cerebral hemisphere supplied by the middle cerebral artery (5). It is more systematic than the one-third middle cerebral artery territory rule and has been reported highly correlated to clinical outcomes. Therefore, many guidelines for early AIS management use an ASPECTS evaluation of ≥6 as an inclusion criterion for intravascular thrombectomy treatment (6, 7). Recent study also suggested patients with lower APECTS scores May still benefit from thrombectomy (8).

Noticeably, even with the convenience of NCCT, the interpretation of core infarct areas can be subjective and highly dependent on the radiologists’ experience (9). A systematic review found that, given the various levels of agreement among clinicians assessing ASPECTS in thrombectomy candidates (ICC 0.672–0.811, kappa 0.042–0.469), the inconsistency is significant enough to question its reliability for treatment decisions (10). The sensitivity of stroke diagnosis by physicians based on NCCT within 24 h is 57–71%, with only 12% in the early 3 h (11). Accurately identifying small infarct foci on NCCT is more challenging in patients with ASPECTS scores ≥6 compared to those with large infarcts (10).

However, the development of artificial intelligence (AI) technology has helped address this issue. Automated software based on machine learning (ML) has been widely used in the differential diagnosis and prognosis prediction of cerebrovascular diseases (12–14). Studies have shown that automatic ASPECTS evaluation programs, such as the e-ASPECTS, RAPID, and Frontier programs, can perform statistically non-inferior, or even equivalent, to experienced neuroradiologists (6, 15, 16). However, these existing automated ASPECTS scoring software employ homogeneous principles in ML methods and rely heavily on comparisons of Hounsfield units (HU) between ipsilateral and contralateral brain regions. This approach has limitations in patients with subtle ischemic changes, as well as when images have low signal-to-noise ratios and motion artifacts (17). Further, some automated ASPECTS models use CT perfusion imaging (CTP) as reference gold standard of infarct core (18, 19), which is controversial due to its low resolution and unreliable precise core infarct foci information (20, 21).

Diffusion MRI, recognized as the gold standard for determining the core of acute ischemic infarction (22), provides the most accurate assessment but is limited by scanning preparation time and MR-related contraindications, restricting its wide application in immediate AIS diagnosis. Therefore, accurate diagnosis of AIS by NCCT and rapid identification of small infarct foci within the effective time window are important for clinicians to select surgical options and predict patient prognosis (23).

In this study, we proposed using YOLOv5 as the basic target detection model to automatically identify the core area of AIS infarcts (especially for small infarcts, i.e., ASPECTS score ≥ 6) on NCCT. By comparing and optimizing the loss function as well as using DWI infarcts (within 2 h after NCCT) as a reference, our network trained the model to achieve higher diagnostic performance. In addition, we compared the diagnostic efficacy of this model to manual assessments using the DWI-ASPECTS scoring system as the standard.

## 2 Methods

### 2.1 Study cohort

In the current study, we retrospectively collected AIS patients who underwent examinations in the radiology department of our hospital from January 2018–2022. Our hospital has established a “green channel” for emergency stroke care, also referred to as a priority pathway, ensuring that patients with acute stroke receive prompt diagnostic and treatment services.

Inclusion criteria: (1) clinical symptoms, signs, and imaging manifestations were consistent with the diagnosis of ischemic stroke (24); (2) completion of NCCT examination with images available for assessment (no obvious motion artifacts, etc.); (3) completion of MRI examination (including DWI, ADC, T2-Flair and 3D-TOF images) with images available for assessment; (4) patients underwent NCCT examinations within 6 h of symptom onset and did not receive any recanalizing/reperfusion treatments, as confirmed by interviews and the attending emergency physician; (5) NCCT and MRI examination interval < 2 h; (6) ASPECTS score ≥ 6.

Exclusion criteria: presence of infarct foci in the posterior circulation blood supply area, incomplete clinical data, patients with intracranial hemorrhage, cranial tumor, post-cranial surgery, and wake-up stroke patients (25).

### 2.2 Image acquisition

#### 2.2.1 Unenhanced CT acquisition

Unenhanced brain CT images were acquired using a Somatom Definition Flash or a Somatom Force CT machine (Siemens Healthcare, Forchheim, Germany). For all examinations, automated tube voltage selection (Care kV, Siemens Healthcare) was used with a quality reference tube voltage of 120 kVp. Automatic tube current modulation (CARE Dose 4D; Siemens Healthcare) was applied with a quality reference tube current time product of 330 mAs (Flash) and 273 mAs (Force).

#### 2.2.2 MRI acquisition

MRI images were scanned on Discovery MR750W 3.0-T MRI scanner (GE Healthcare, Waukesha, WI, United States) with a 16-channel head/neck combined coils. DWI images were obtained with the following parameters: TR = 4,300 ms, TE = 109 ms, averages = 2; acquisition matrix = 192 × 192, slice thickness = 6 mm, and slice =16. 3D TOF-MRA images were obtained with parameters of TR = 19 ms, TE = 3.6 ms, averages = 1, FOV = 220 mm × 80.5 mm, acquisition matrix = 512 × 512; slice thickness = 0.7 mm. The T2-weighted FLAIR sequences were: TR = 7,000 ms, TE = 94 ms; averages = 1; FOV = 220 mm × 100 mm; acquisition matrix = 256 × 256; slice thickness = 6 mm, slice =6.

### 2.3 Image analysis

#### 2.3.1 Image analysis by neuroradiologists

According to the ASPECTS criteria, all unenhanced brain CT images within 6 h of symptom onset were reviewed retrospectively and independently by two board-certified neuroradiology physicians (with 25 and 26 years of experience, respectively). Basic clinical information or lateralization of symptoms/stroke were disclosed to neuroradiologists. Final consensus of the AIS infarct core diagnosis would be achieved after discussion of two physicians. The manual diagnosis of CT-ASPECTS would be compared to the reference standard scores that based on the ASPECTS scores summarized from DWI images observed by two radiologists (31 and 32 years of experience, respectively).

#### 2.3.2 Image preprocessing

All CT images displaying AIS core infarcts were manually labeled by radiologists using Labelme software. All CT images were assigned to two board-certified radiologists to delineate ground truth via consensus. A third board-certified radiologist was consulted in cases of disagreement.

Initially, the DWI images were registered to the corresponding CT images using the Dual Attention VoxelMorph Network (26), which offers enhanced registration accuracy and model sensitivity with minimal computational overhead. After registration, radiologists labeled the core infarcts on the annotated CT images while referring to the registered CT-DWI images.

We applied a series of preprocessing steps to the NCCT images. These steps included standardizing the resolution of all images to 640 × 640 pixels, normalizing the image contrast using brain window settings (window level: 30 HU, window width: 60 HU) for all head NCCT images, applying z-score normalization, and augmenting the data through random horizontal flipping and cropping.

The 10 paired ASPECTS regions from the preprocessed CT images were segmented using the widely employed V-net architecture (27). V-net has been extensively utilized for medical image segmentation, particularly in brain tissue segmentation (28, 29).

#### 2.3.3 Image analysis by proposed model

**Figure 1:**
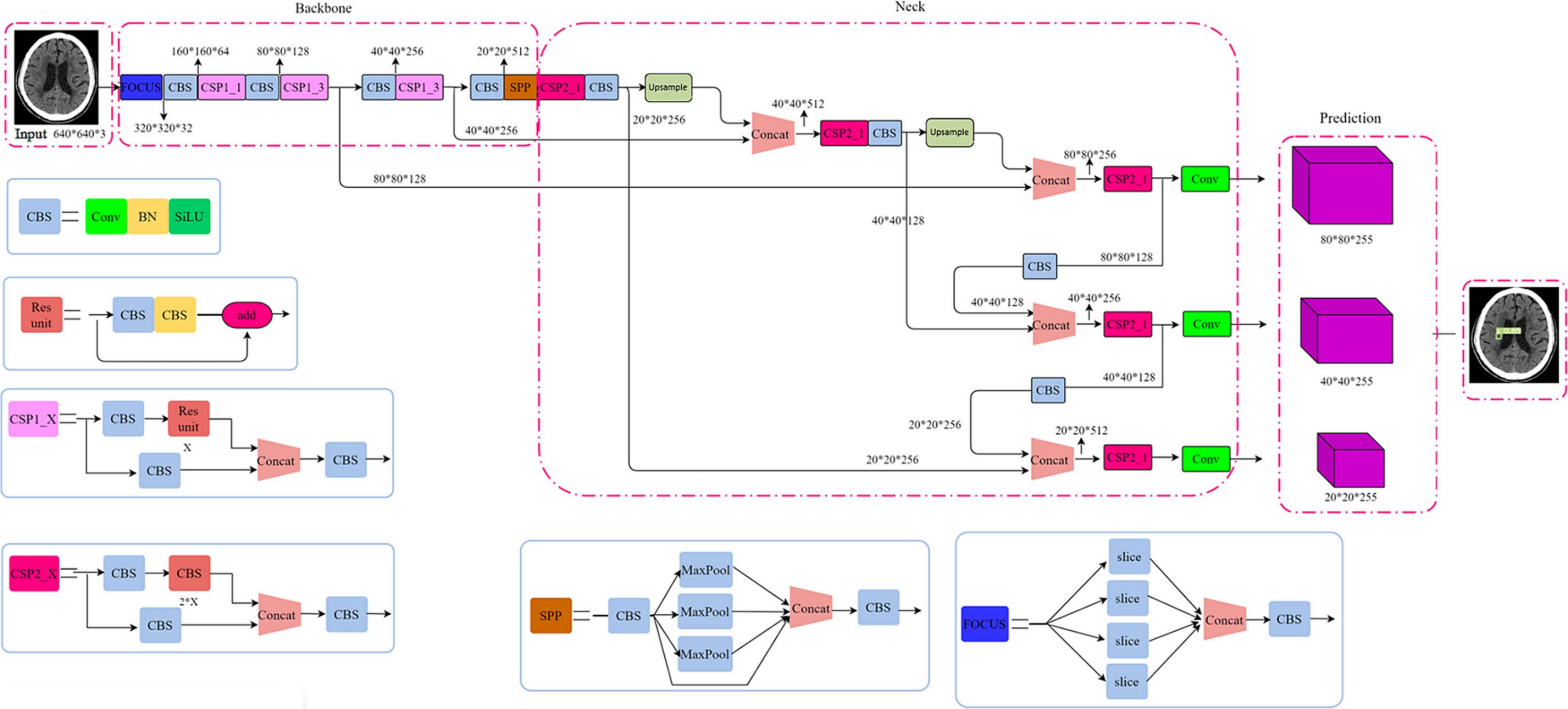
The structure of the YOLOv5 model.

Our proposed model adopted a single-stage detection method from YOLOv5 (30). Shown as Figure 1, the structure of YOLOv5 model consists of backbone, neck and head. Cross Stage Partial Networks (CSPNet) based on DenseNet architecture were used as the backbone to extract rich and useful features from an input image. The neck included PANet and SPP, where SPP (spatial pyramid pooling) enhances the model’s detection of objects with different scales and PANet (Path aggregation network) is the neck for feature aggregation (30) and to generate feature pyramids. The head model is used for final inspection, where anchor boxes are used to feature the map and generate the final output vector with class probabilities, object scores, and bound boxes.

Although the YOLOv5 reports high inference speed and small model sizes to allow a convenient translation to mobile use cases via model export (31), the imbalance of positive and negative samples in this study restricts the application of basic YOLOv5 model. We modified the R-Mish activation function and intersection over union (IoU) loss function (32, 33) of YOLOv5 to detect infarct cores correctly even on the complex surfaces. Specific details will be disclosed in the Supplementary materials.

For constructing baseline models, we employed a workstation with 11400F CPU, 32GB system memory and NVIDIA RTX3070GPU with 8GB memory. Both stages used the default set of hyper parameters (including parameters related to the data augmentation procedures) offered by the YOLO framework, which was based on Pytorch framework. We used different bounding box loss functions for testing. The parameters were modified based on the current data. The main parameter in this paper were: the number of image categories, the number of training times is based on 100, appropriately increased to 300, and the size of the training image: 512*512. Before training we set the threshold value to 0.5, that is, the result predicted by the neural network model in the training process is greater than this value can be regarded as a positive sample, and vice versa as a negative sample. After a certain number of training sessions, the model with the best training result will be obtained, which will be used to assist in finding the lesion area in CT images. The mean Average Precision (mAP%) is used as a reference indicator for good model training results.

### 2.4 Statistical analysis

Normality distribution data was assessed by Kolmogorove-Smirnov test (*α* = 0.05). Otherwise, medians, interquartile ranges, and ranges are given. The agreement among neuroradiologists, model and reference standard of DWI on the ASPECTS evaluation was calculated by means of the intraclass correlation coefficient (ICC) and weighted kappa. The sensitivity, specificity and accuracy of each subregion (M1-M6, insula, lenticular nucleus, caudate nucleus, and internal capsule) in the ASPECTS scoring system was calculated by confusion matrix. To assess the models’ performance, we used the IoU metric to measure overlap between predicted and ground truth boxes. A detection was considered correct (True Positive, TP) if the IoU exceeded 0.5; otherwise, it was classified as a False Negative (FN). The IoU threshold was set at 0.5. In the ASPECTS scoring system, if the model detected a lesion with a probability greater than 0.5, one point was subtracted from the maximum score of 10 for that region. The paired chi square test was used to compare the diagnostic efficacy of physician groups and automated models in subregions. *p* < 0.05 was considered statistically significant.

## 3 Results

### 3.1 Demographics of the study cohort

Between 2018 and 2022, in a total of 584 AIS patients underwent examinations in the radiology department. However, only 275 cases were eligible for the current study, the exclusion cases were: 25 cases of motion artifacts, 78 cases that core infarct area accumulated in the posterior circulation area, 71 cases that stroke symptoms occurred more than 6 h before coming to the hospital for NCCT, 43 cases that the interval between NCCT and MRI examination was more than 2 h, 55 cases with large infarcts due to postoperative or incomplete medical records, and 37 cases with ASPECTS score < 6.

The 275 cases were completely randomly divided into a training set and an independent testing set at a 10:1 ratio. Clinical and imaging indices were shown in the Table 1.

**Table 1:** Patient’s clinical record in the training and testing tests.

Category	Training set(250 cases)	Testing set(25 cases)
Average age (years)	68.08 ± 11.70	67.16 ± 9.38
Male [n (%)]	172(68.8)	19(76.00)
History of ischemic stroke [cases (%)]	139(55.6)	15(60.00)
Hypertension [cases (%)]	120(48.0)	19(76.00)
Diabetes [cases (%)]	157(62.8)	15(60.00)
Smoking history [n (%)]	147(58.8)	18(72.00)
Mean time to onset of stroke from NCCT examination (h)	3.85 ± 1.82	3.68 ± 1.15
NCCT interval from MRI examination<1 h [n (%)]	131(52.4)	9(36.00)
NCCT interval from MRI examination1^2h [n (%)]	119(47.6)	16(64.00)
DWI-ASPECTS [M (P25, P75), scores]	8(7, 8)	8(7, 9)

### 3.2 Outcomes of the deep learning recognition of core infarct area

**Figure 2:**
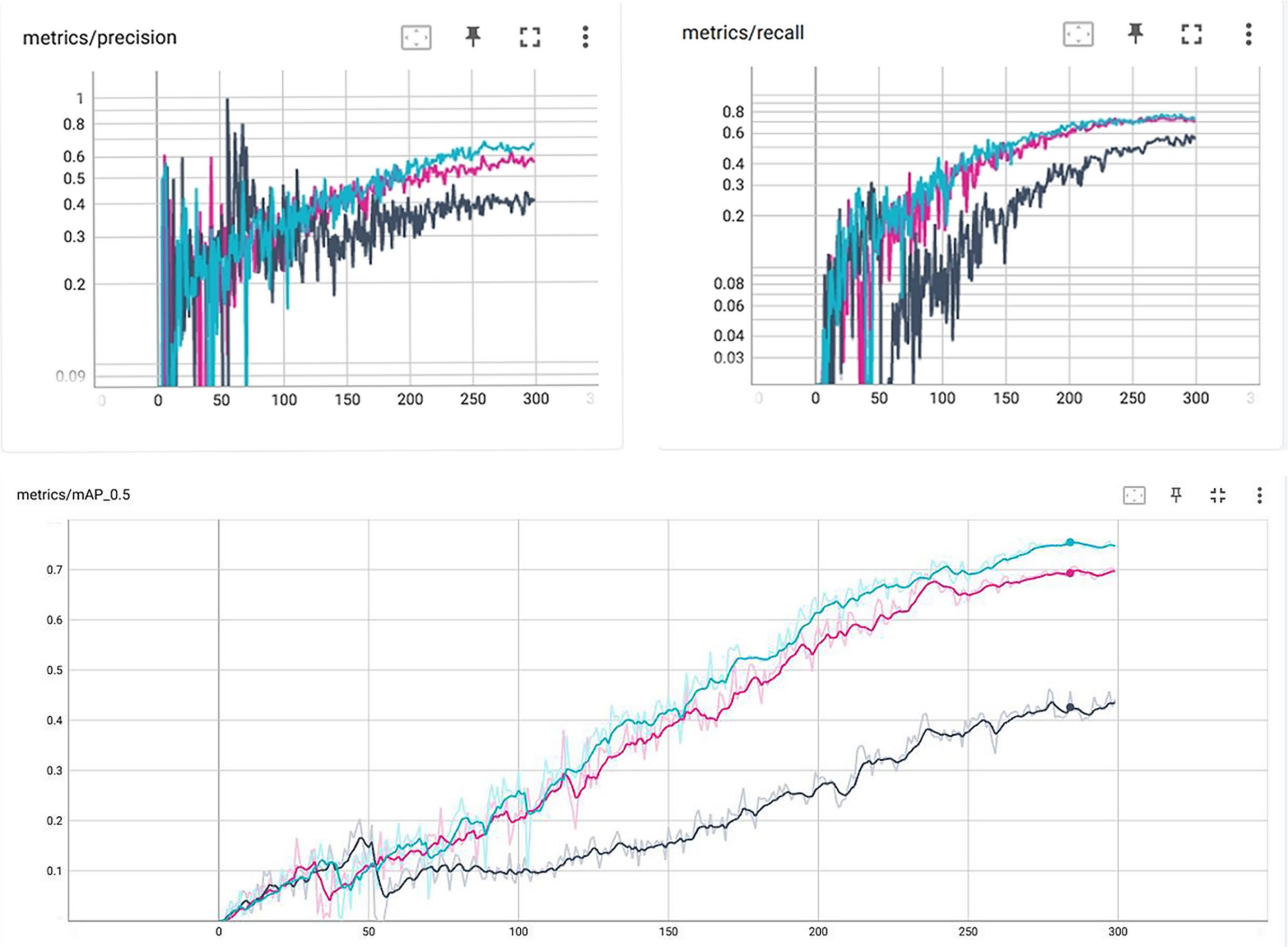
Parameter change curves for different loss functions during the training process. M-CIoU is in color blue, CIoU is in color pink and the DIoU is in gray.

After experimental testing, our modified complete IoU (M-CIoU) module had the best training outcomes than the CIoU and DIoU (Distance IoU) loss function. Detailed results were shown in the Figure 2 and Table 2.

**Table 2:** Testing results of three different loss function.

	CIoU (pink)	DIou (gray)	M-CIoU (blue)
Precision	0.616	0.8014	0.8237
Recall	0.7377	0.5942	0.8101
IoU (map@0.5)	0.706	0.4629	0.7851

### 3.3 Results of consistency analysis

The ICC between our model diagnosed ASPECTS scores and DWI ASPECTS scores was 0.669 (95% interval [CI] 0.380–0.839, *p* < 0.001). The ICC between physician scored ASPECTS and DWI ASPECTS was 0.452 (95% interval [CI] 0.077–0.715, *p* = 0.010).

The consistency testing between our model scored ASPECTS and the DWI ASPECTS achieved kappa of 0.477 (95% interval [CI] 0.255–0.699, *p* < 0.001). The consistency between physician scores and the DWI ASPECTS achieved the kappa of 0.247 (95% interval [CI] −0.017–0.510, *p* = 0.054).

In addition, the sensitivity, specificity, and accuracy results of three different diagnoses of AIS were shown in the Table 3. The paired Chi-square test showed that the automatic model had better diagnostic efficacy than the physician diagnosis in the M6 region (*p* = 0.039).

**Table 3:** Sensitivity, specificity and accuracy of the CT manual ASPECTS scoring system.

	Sensitivity (%)		Specificity (%)		Accuracy (%)	
	Model	Physician	Model	Physician	Model	Physician
M1	33.30	66.67	100.00	93.62	96.00	92.00
M2	80.00	40.00	100.00	97.78	98.00	92.00
M3	100.00	75.00	97.83	97.83	98.00	96.00
M4	50.00	83.33	100.00	90.91	94.00	90.00
M5	90.00	75.00	96.67	93.33	94.00	86.00
M6	85.71	28.57	100.00	93.02	98.00	84.00
Insula	33.33	33.33	97.87	93.62	94.00	90.00
Lenticular nucleus	100.00	100.00	100.00	100.00	100.00	100.00
Caudate	50.00	100.00	100.00	100.00	98.00	100.00
Internal capsule	66.67	50.00	95.45	88.64	92.00	84.00
Total	75.86	63.79	98.87	95.02	96.20	91.40

### 3.4 Case demonstration

**Figure 3:**
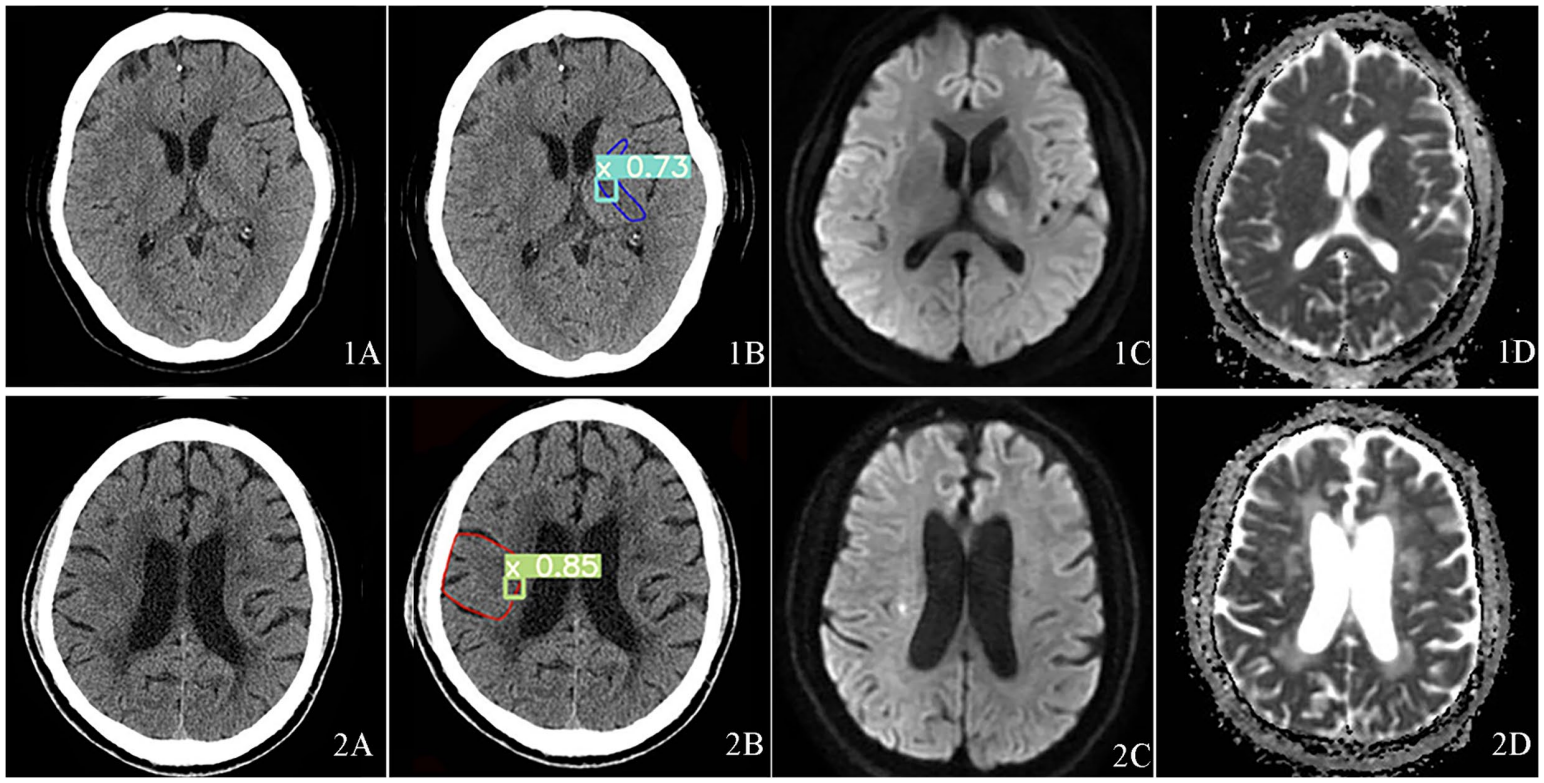
Visualization of acute core infarct recognition on NCCT images using the deep learning model for case 1 and case 2. Case 1 was shown at the top and Case 2 at the bottom. Case 1 and 2 images were shown as NCCT (labeled as **1A** and **2A**), automatic model diagnosed images (labeled as **1B** and **2B**), DWI images (labeled as **1C** and **2C**) and ADC images(labeled as **1D** and **2D**).

Case 1, 2 were typical cases in the missed cases during physicians’ diagnoses, indicating the difficulty of recognition of small core infarct area (Figure 3).

In case 1, the ASPECTS score was 9, the lesion was located on the hind limb of the left internal capsule. Figures 3-1A was the NCCT. Figures 3-1B was the automatic diagnosed figure, where the green box indicated the core infarct area, with a 73% probability of the area being an acute infarct. Figures 3-1C was the DWI image, and the Figures 3-1D was the ADC image.

In case 2, the ASPECTS score was 9, and the lesion location was M5. Figures 3-2A was the NCCT, Figures 3-2B showed the automatic diagnosed image, where the green box indicted the identified core infarct area with an 85% of probability of the area being an acute infarct. Figures 3-2C and Figures 3-2D showed the DWI image and ADC image, respectively.

**Figure 4:**
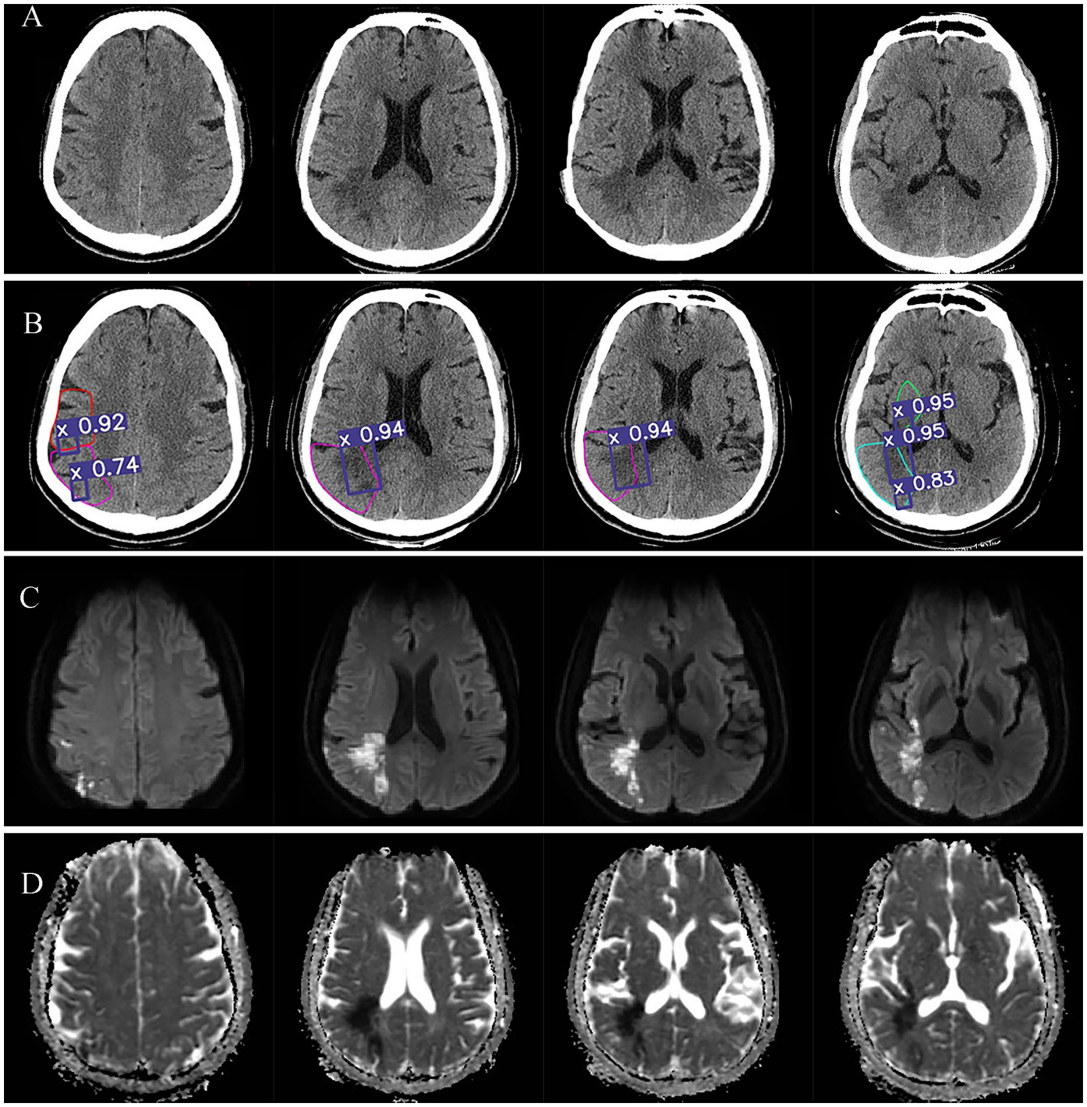
Visualization of acute core infarct recognition on NCCT images using the deep learning model for case 3. Case 3 images were shown as NCCT (labeled as **A**), automatic model diagnosed images (labeled as **B**), DWI images (labeled as **C**) and ADC images (labeled as **D**).

Figure 4 illustrates Case 3, which showed mixed foci with the infarct extent spanning two brain regions simultaneously, and an ASPECTS score of 6 with lesion locations in M2, M3,M5, and M6. The Figure 4A showed the NCCT image. Figure 4B showed the automatic diagnosed images, where the purple boxes demonstrated the core infarcts areas, with the 92, 74, 94, 95, and 83% of probabilities of being acute infarcts areas indicated the acute infarcts probabilities, respectively. Figure 4C was DWI image and Figure 4D as ADC image.

## 4 Discussion

**Figure 5:**
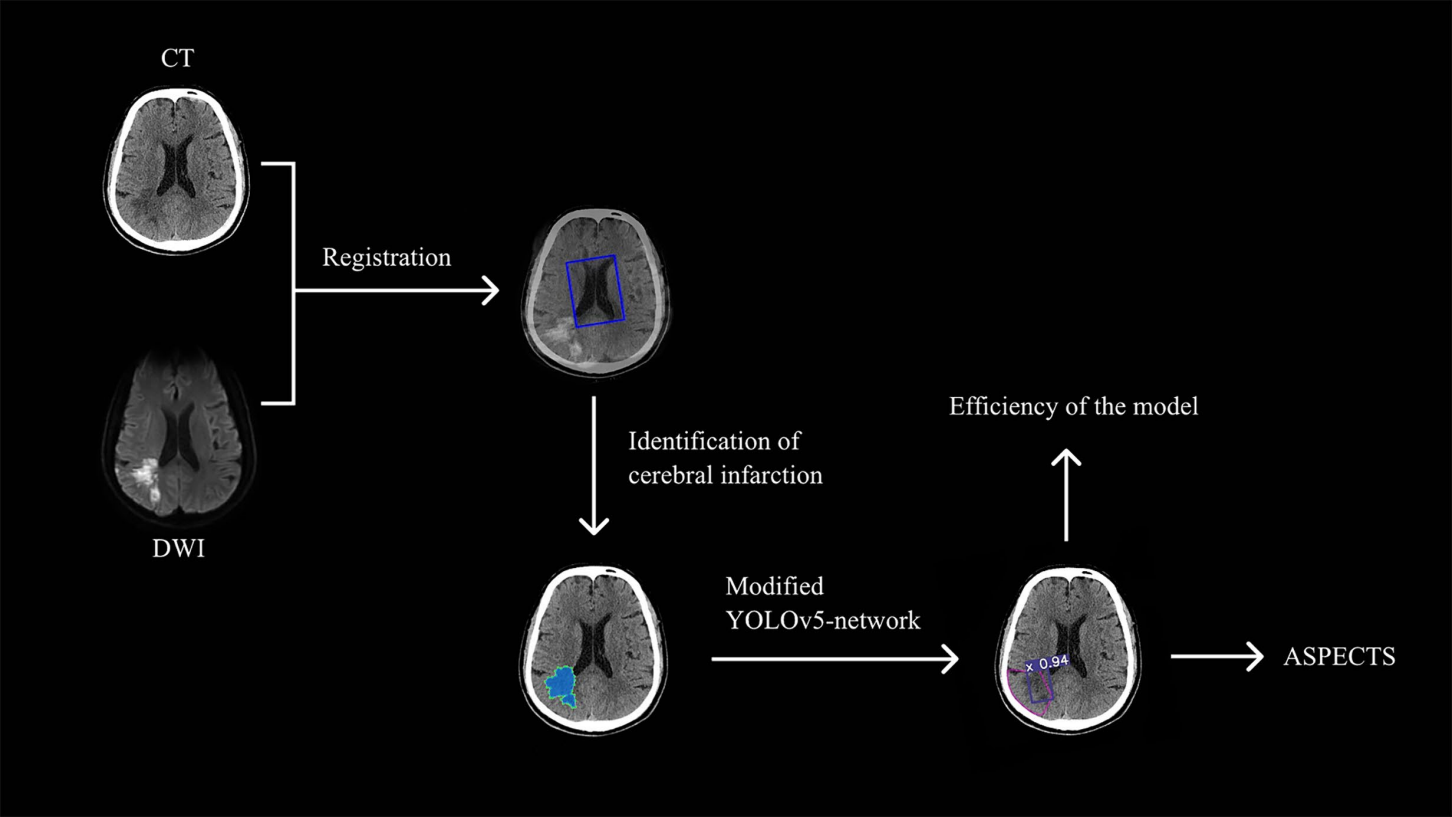
The technical pipeline of the study.The study begins with the registration of CT and DWI images using the Dual Attention VoxelMorph Network. Radiologists then labeled the core infarcts on the CT images, which were subsequently used to train the Modified YOLOv5-based model. Finally, the trained model efficiently recognizes acute infarct cores and calculates the ASPECTS score.

This study aimed to employ a target-based deep learning network to improve the accuracy of NCCT based AIS diagnosis so to identify small infarct foci within the effective time window. The results showed our proposed modified YOLOv5 model is a fast and compact object detection model for automated identification of AIS infarct core area on NCCT images. Compared to the traditional physician interpretation, which takes a few minutes, the proposed automated model reads AIS in only 10 s (23). In addition, the ASPECTS scores of the automated recognition model showed higher consistency to the DWI-ASPECTS scores than the ASPECTS scores graded by the physician group. The diagnostic efficacy of multiple regions was higher than that of the physician group, especially for small areas of acute cerebral infarction (Figure 5).

Our proposed model showed a superior consistency with the reference standard score (ICC = 0.669, *κ* = 0.447) than neuroradiologists (ICC = 0.452, κ = 0.247) who had particular expertise in evaluating diagnostic studies in acute stroke patients, also the mean sensitivity, specificity, and accuracy of nearly every subregion were better than neuroradiologists. Previous studies have confirmed the challenge of detection of subtle signs of early ischemia for even experienced physicians (34). Prior analytic results showed that ASPECTS achieved insufficient agreement between clinicians for ASPECTS to be reliably used as a criterion for treatment decisions, inter-rater agreement was slight to moderate (κ = 0.129–0.315). Even in the best of cases, when ASPECTS was dichotomized as 0–5 vs. 6–10, interrater agreement did not reach a substantial level (κ = 0.561) (10). Wilson AT et al. point out that as the development of automated computational tools to assess ASPECTS on NCCT, the inter-rater reliability issue May become less pertinent than the issue of human versus computer performance (35). The detection rate of AIS lesions within 3 Hours of onset was low by neurologists and CT-ASPECTS scores could not predict the favorable and non-favorable outcome groups (36).

The present study chose DL algorithms for a reason. The drawbacks of ML based software cannot be neglected. For example, there are several popular commercial software in the clinical settings. Studies have shown software programs such as e-ASPECTS, RAPID and syngo.via Frontier ASPECTS prototype can be statistically non-inferior or equivalent to three independent neuroradiologists (NRADs) when diagnosing early ischemic damage (15, 16). These programs used ML highly depend on first-order image features to discover the presence of infarct foci in certain regions, such as Hounsfield unit (HU) or density (6). These first-order image features have limitations in patients with subtle ischemic changes and when images have low signal-to-noise ratios and motion artifacts (17). Additionally, ML-based approaches greatly rely on comparisons of the ipsilateral and contralateral brain regions, as this is how humans interpret images (17). This would result in low sensitivity. The e-ASPECTS software revealed such low sensitivity in both two studies, 44 and 46.46%, respectively (15, 37). Although the RAPID showed the best agreement to the consensus score (k = 0.9), its restricted application cannot be ignored. When patients with ASPECTS score ≥ 7, the probability of detecting infarction by RAPID decreased substantially. Only about 20% (22 of 100 in cohort 1, 10 of 52 in cohort 2) of the CT data sets could not be analyzed by using RAPID (6).

Given the aforementioned limitations of ML models, more recent studies have optimized the extraction of features in the ML models. For example, Kuang et al. introduced multiple high order computational textural features into the ML model and showed a more accurate and reliable ASPECTS reading compared to that on acute DWI (17). This method used nonlinear self-registration to correct brain asymmetry and calculated bilateral differences at different scales to tolerate suboptimal symmetry. However, it would result in the increasing variability and complexity of ML model and May introduce unpredictability into their model. Therefore, the current models based on ML algorithms cannot overcome the defects, such as poor generalization ability. So that the diagnosis is more unreliable when the case is an acute ischemic stroke in bilateral brain regions.

Notably, the major strengths of convolutional neural network (CNN) from DL can overcome the weak contrast of the infarct tissue in the early stage and naked eye’s low ability in detecting subtle differences. The modified target detection method proposed in the current study can acquire and learn high-dimensional information of images directly from the data. The algorithm not only gives the detected target class, but the location and range of lesion. Among object detection models such as faster R-CNN and YOLO, we chose to use YOLO for the following reasons. Firstly, it is well recognized that realizing CNN feature extraction on each candidate frame and calculations would take up a large amount of memory space and overlaps. YOLO model can be superior in the detection speed. It uses the entire image as the input to the network to convert detection problem into a single regression problem and directly return the target frame of its position in multiple positions of the image and the category to which the target belongs (38). Secondly, YOLOv5 has demonstrated reliable detection performance with an overall high precision over different model configurations (31). The present study proposed a lightweight detection and classification method based on modified YOLOv5 to detect infarct core of AIS. Improvements of activation function R-Mish and CIoU loss have showed superiority in the detection of lesions, especially in our dataset that had fewer positive samples. The results showed that modified versions of YOLOv5 perform better with incensed precision (0.82), recall (0.81) and mAP (0.79) than original YOLOv5. Due to its reasonable performance and rapid end-to-end technique for detecting objects, our model only takes a few seconds to detect a single case, which is much faster than the 5-min reported previously (12). Moreover, our model is small and can be easily transplanted on mobile devices, making it applicable in other fields.

Our results reported that our proposed model can quickly identify small cerebral infarcts that could not be quickly detected by some physicians at NCCT (shown as Figures 3, 4). The better performance of this model can be explained by the subtle changes corresponding to DWI image. Our model favorably detected subtle changes without follow-up imaging procedures. Our observations can be surpassing many previous studies. For example, Pan et al. used a DL residual network (ResNet) to detect the infarct core on NCCT images based on DWI to improve the accuracy of acute ischemic stroke diagnosis (39). However, the insufficient number of cases and absence of ASPECTS areas segmentation making it difficult to evaluate its clinical applicability. Barros et al. used separate three CNNs for the segmentation of the final infarct on follow-up NCCT scans and reported an excellent agreement with the manually reference with an ICC of 0.88 (40). However, the absence of gold-standard makes it impossible to identify the early ischemic injury. Same problem also existed in some other studies (41), where using experts’ manual contouring as the reference standard can cause considerable observer variability. For data-driven approach, the uncertainty and variability of reference standard May introduce unpredictability into the DL model, thereby increasing the complexity of the problem. Although some DL models tried to construct the gold standard for CTP, it remains controversial to use CTP to determine the infarct core due to is low resolution and debatable cutoff value, and lacking of reliable information about the precise core infarct foci (20, 21). A recent study used CTP to detect ischemic regions for ASPECTS scoring. It designed a depth-asymmetric network (DA-Net) on an asymmetric structure to detect differences between the left and right hemispheres in order to estimate their ischemic status (18). Accordingly, it can automatically calculate the ASPECTS score. Due to the lack of DWI reference for the core infarct region, they mainly evaluate the performance of estimating whether the ASPECTS score is higher than 6. In addition, this asymmetric network model for determining ischemic brain regions does not visualize the core infarct foci, which provides limited assistance to the imaging physician when performing image analysis.

Therefore, our proposed model included spatial information to improve the performance in detecting small focal lesions. The gold standard is built based on the MR (DWI, T2-FLAIR, 3D-TOF, ADC) within 2 h after NCCT. DWI is a highly sensitive MRI technique that can provide more reliable information for AIS such as core infarct area and infarct extent, and is relatively better able to distinguish the infarct core from the old lesions (42).

The present study used AIS patients for retrospective analysis, from which the patient acute state is defined as patients within 6 h after onset of stroke symptoms. This period is important since assessing the extent of infarction within 6 h is critical for determining the most appropriate treatment strategies moving forward (7). Images included in the dataset of this study were heterogeneous, including images of different manufacturers, parameters, and layer thicknesses. It is noticeable that we obtained higher accuracy but lower average sensitivity in the detection and analysis of sub-regions. The sensitivity of M1, M4 and insula was lower than 50%. This was mainly caused by the small number of positive samples are in these subregions. Since we adopted the subregion-based detection model, number of positive samples in these areas in the test set is small and some misjudged samples will appear. False negative identification results would appear due to some reasons such as insufficient data set of the model. The model in the present study could still provide more reference information to the imaging physicians and save the diagnosis time. The rate of missed diagnosis can be significantly reduced.

In addition, this study screened the dataset was targeted to include only small infarcts with ASPECTS scores ≥6 to test the model, so it was more challenging in terms of diagnostic difficulty, but the diagnostic efficacy of the automated model in this study in terms of consistency (ICC = 0.669), total sensitivity (75.86%), total specificity (98.87%) and total accuracy (96.20%) has been comparable to current models that do not target small cerebral infarction. We selected patients with ASPECTS scores ≥6 to align with current clinical guidelines for interventions like mechanical thrombectomy (7). This focus ensures clinical relevance but May limit applicability to patients with lower scores. Future studies should include a wider range of ASPECTS scores to assess the model’s performance across different patient populations.

There are several limits in the present study. First, the proposed model is only trained to test the MCA blood supply area, and acute ischemic stroke in the posterior circulation is more difficult to detect these lesions than by anterior circulation stroke due to ray-hardening artifacts, so further research is needed. Secondly, the number of cases is relatively small. To improve the robustness and generalization ability of the algorithm, we also need to further build a feedback mode for clinical practice. A feedback mode could realize the software data polycentric so that to improve the software accuracy. Last but not the least, NCCT images can be used for rapid identification of a variety of diseases, including ischemic, hemorrhagic cerebrovascular disease, large vessel occlusion, and can rapidly assess the ischemic semi dark zones by CT. If the future model can recognize stroke types, it would be more beneficial.

## 5 Conclusion

In this paper, we construct a deep learning target detection network-based model for automated identification of infarct cores in acute ischemic stroke in NCCT by optimizing the loss function of target detection model using the gold standard of DWI. The results showed that the model was more effective than the physician group in identifying the infarct core in the acute phase of AIS patients, especially for small areas of acute cerebral infarction (ASPECTS score ≥ 6). The modified YOLOv5 achieved better diagnostic performance and accuracy than original version. The deep learning network based on target detection not only gives more accurate ASPECTS scores, but also provides a simple and intuitive understanding of the infarct portion and extent, which is valuable in assessing the severity of disease and selecting treatment procedures. Due to its reasonable performance and rapid end-to-end technique for detecting, this model only takes a few seconds to detect a single case which should help clinicians optimize the process of cerebrovascular disease and reduce the rate of missed diagnoses more effectively.

## Data Availability

The raw data supporting the conclusions of this article will be made available by the authors, without undue reservation.
