# Supplementary material for: Target-based deep learning network surveillance of non-contrast computed tomography for small infarct core of acute ischemic stroke
Source: Front Neurol. 2024 Sep 19;15:1477811. doi: 10.3389/fneur.2024.1477811 (PMC11447964; doi:10.3389/fneur.2024.1477811)
Supplement: Supplementary file 1 [file Presentation_1.pdf]

Supplementary Material

Supplementary Data

Modification of YOLOv5:

Noted that activation function acts important role in deep learning networks, we designed a segmented R-Mish activation function based on Mish and ReLU activation functions. Equations are listed below from ReLU (1), Mish (2), and our modified R-Mish activation functions (3).

In the YOLOv5 model, the head model is the same as the previous Yolov3 and Yolov4, which is mainly used in the final inspection part. It applies anchor boxes to the feature map and generates the final output vector with class probabilities, object scores and bounding boxes.

$$Relu = \max(0, x) \tag{1}$$

$$Mish = h(x) = x * \tanh(\ln(1 + \exp(x))) \tag{2}$$

$$R-Mish = \begin{cases} Mish & x \leq 0 \\ \mathrm{Re}lu + \sin(\frac{epsilon}{x}) & x > 0 \end{cases} \tag{3}$$

The Leaky Relu activation function in the YOLOv5 is used in the middle/hidden layer. The Sigmoid activation function is used in the same segmented design as Relu. For the negative part in the R-Mish function, the R-Mish equals to the Mish. Because Mish itself is a non-monotonic function, where negative values have gradients. For the positive part, R-Mish and the Relu are similar, but we added the $\sin(\frac{epsilon}{x})$ to guarantee the smoothness and increase the nonlinearity to fit the model.

Secondly, we modified the CIoU (Complete Intersection over Union) loss function, which originally proposed by Zheng et al. targeted at the bounding box position regression problem [1]. The loss function is an important indicator to measure the generalization ability of the model. We trained this model by calculating the gap between the predicted value and the true value of the data. The ultimate goal of optimizing the model was to reduce the loss value as much as possible without

fitting. YOLOv5 uses the following GIoU_Loss as the loss function of the bounding box. Given the bounding box regression is the critical part in target detection, many loss functions have been proposed to increase the sensitivity of the model. Differentiated from Intersection over Union (IoU), GIoU (Generalized IoU), and DIoU (Distance IoU), CIoU can effectively improve the convergence speed and accuracy of regression by merging the normalized distances between the prediction and target frames [2]. Our modified loss function shows as below:

$$\Gamma_{CIoU} = 1 - IoU + \frac{\rho^2(b, b^{gt})}{c^2} + \alpha v \tag{4}$$

$$\alpha = \frac{v}{(1 - IoU) + v} \tag{5}$$

$$v = \frac{4}{\pi^2}(\arctan\frac{w^{gt}}{h^{gt}} - \arctan\frac{w}{h})^2 \tag{6}$$

From which, gt denotes the real frame, IoU denotes the ratio of the overlapping area of the real frame and predictive frame. $\rho^2(b, b^{gt})$ denotes the square of the distance between the center of the prediction frame and the real frame, c denotes the square of the diagonal distance between the prediction frame and the real frame, ν is the parameter describing the consistency of the aspect ratio, and α is its penalty parameter.

Based on the characteristics of the positive and negative samples, we modified the equation (5) and had the new penalty function as below (equations 7 and 8):

$$v = \frac{4}{\pi^2}(\varphi(\frac{w^{gt}}{h^{gt}}) - \varphi(\frac{w}{h}))^2 \tag{7}$$

$$\varphi(x) = \frac{1}{1 + e^{-x}} \tag{8}$$

Reference:

1. Zheng, Z., Wang, P., Liu, W., Li, J., Ye, R., & Ren, D. (2020). Distance-IoU Loss: Faster and Better Learning for Bounding Box Regression. Proceedings of the AAAI Conference on Artificial Intelligence, 34(07), 12993-13000. doi: 10.1609/aaai.v34i07.6999

2. He, K., Zhang, X., Ren, S., & Sun, J. (2015). Spatial pyramid pooling in deep convolutional networks for visual recognition. IEEE Transactions on Pattern Analysis and Machine Intelligence, 37(9), 1904-1916. doi: 10.1109/TPAMI.2015.2389824
